# Genetic analysis and comparative virulence of infectious salmon anemia virus (ISAV) types HPR7a and HPR7b from recent field outbreaks in Chile

**DOI:** 10.1186/s12985-014-0204-1

**Published:** 2014-11-29

**Authors:** Marcos G Godoy, Rudy Suarez, Eduardo S Lazo, Katerina O Llegues, Molly JT Kibenge, Yingwei Wang, Frederick SB Kibenge

**Affiliations:** Centro de Investigaciones Biológicas Aplicadas (CIBA), Diego de Almagro Norte 1013, No. 10, Puerto Montt, Chile; Facultad de Medicina Veterinaria, Universidad San Sebastian, Lago Panguipulli 1390, Puerto Montt, Chile; ETECMA, Diego de Almagro Norte 1013, No. 10, Puerto Montt, Chile; Department of Pathology and Microbiology, Atlantic Veterinary College, University of Prince Edward Island, 550 University Avenue, Charlottetown, P.E.I., C1A 4P3 Canada; Department of Computer Science and Information Technology, University of Prince Edward Island, 550 University Avenue, Charlottetown, P.E.I., C1A 4P3 Canada

**Keywords:** *Salmo salar*, Infectious salmon anemia virus, Virulence markers, ISAV mutations, ISAV clades

## Abstract

**Background:**

Infectious salmon anemia (ISA) is a serious disease of marine farmed Atlantic salmon, *Salmo salar* L. caused by ISA virus (ISAV). ISAV genomic segments 5 and 6 encode surface glycoproteins hemagglutinin-esterase (HE) and F protein important for the pathogenicity of ISAV. In this study, we describe the genetic characteristics and relationship between ISAV-HPR7a and ISAV-HPR7b strains that caused the ISA outbreaks in Chile in 2013 and 2014, respectively, and the evolution of the ISAV clades since 2009 based on segment 5 and 6 sequences.

**Methods:**

The study material included samples from six ISA cases in Chile. RNA was extracted from salmon tissues and ISAV isolated from cell culture; segments 5 and 6 were amplified by RT-PCR and compared by alignment with ISAV sequences from the GenBank database.

**Results:**

ISAV-HPR7a and ISAV-HPR7b belong to the European Genotype I strains only found in Europe and Chile, and in both cases, show high similarity in segments 5 and 6 with identity between 95–96%. Our data confirm the hypothesis that the original virus was introduced to Chile in 1996. Compared to the 2007 ISAV-HPR7b isolate, the 2014 ISAV-HPR7b does not have an insertion in segment 5 and was associated with low mortality, which suggests that ISAV virulence was attenuated by the absence of the insertion in segment 5. In contrast, the highly virulent ISAV-HPR14 from April 2013 outbreak did not have the insertion in segment 5 either.

**Conclusion:**

Variability in the ISAV virulence markers supports the quasispecies theory that multiple evolution forces are likely to shape ISAV genetic diversity. Our findings provide evidence of continuing evolution of ISAV in the Chilean aquaculture industry.

**Electronic supplementary material:**

The online version of this article (doi:10.1186/s12985-014-0204-1) contains supplementary material, which is available to authorized users.

## Introduction

Infectious salmon anemia (ISA) is a serious viral disease of marine farmed Atlantic salmon, *Salmo salar* L. caused by ISA virus (ISAV), which belongs to the genus *Isavirus*, family *Orthomyxoviridae* [1]. The mortality in marine fish net-cages rises slowly and can vary from 0 to 90%. In fact, the virus can be present in the net-cage for up to 6 months before significant mortality is noted. ISA is arguably the most important viral disease of marine-farmed Atlantic salmon because of the associated socio-economic losses, and ISAV remains an emerging fish pathogen because of the asymptomatic infections in wild and farmed fish and the potential for emergence of new epizootic strains.

The ISAV genome consists of eight single-stranded RNA segments [1]; sequence analysis of these segments from different ISAV isolates consistently reveals two genotypes designated according to their geographic origin as European (Genotype I) and North American (Genotype II) [2]. Genotype I can be further subdivided into Genogroup 1 (European–in-North America, EU-in-NA) for Genotype I strains also found in North America, and Genogroup 2 (Real European, Real-EU) for Genotype I strains only found in Europe and Chile [3]. ISAV of European genotype can also be differentiated into three genogroups, EU-G1, EU-G2, and EU-G3; the EU-in-NA strains are placed in EU-G2 group, and Real European strains are divided between EU-G1 and EU-G3 groups [4].

ISAV genomic segments 5 and 6 encode two major surface glycoproteins that are believed to be important for the pathogenicity of ISAV. Hemagglutinin-esterase (HE) encoded by segment 6 has receptor-destroying activity and is responsible for receptor binding and virus release [5-7], while the fusion (F) protein encoded by segment 5 is responsible for the fusion of the viral and cellular membranes during virus entry [8]. The systemic ISA is caused by virulent ISAV strains with deletions in HE hyper polymorphic region (HPR) spanning residues V^337^ to M^372^ in the stem adjacent to the transmembrane portion of the protein; the virus is therefore designated as ISAV-HPRΔ. For both European and North American genotypes, a direct functional relationship can be demonstrated between the length of HE protein stem, ISAV cytopathogenicity in cell culture, and pathogenicity for Atlantic salmon [3]. All ISAV-HPRΔ isolates also have either a sequence insertion or Q^266^ → L^266^ mutation in the F protein [3,9,10]. A single report of ISAV-HPR7b with Q^266^ in the F protein provided no sequence data to authenticate it [11]. To date, four types of sequence insertions (IN1 to IN4) have been reported in the F protein: IN1 to IN3 have been detected in Norwegian isolates and IN4 only in Chilean isolates [3].

Non-cultivable, non-pathogenic ISAV detectable only by RT-PCR has full-length HPR sequence (35 amino acids) in the HE-encoding gene and is designated ISAV-HPR0 [4,10,12-19]. All ISAV-HPR0 isolates are characterized by the wild-type F protein with Q^266^ and no sequence insertion [10]. ISAV-HPR0 replicates mainly in the gills causing only transient subclinical infection [16]. This virus has been detected in apparently healthy wild and farmed Atlantic salmon in most regions with Atlantic salmon aquaculture [20], and is considered to have ancestral relationship with ISAV-HPRΔ [21]. Most recently, ISA cases directly linked to the presence of endemic ISAV-HPR0 have been reported [20], supporting the notion that under appropriate conditions ISAV-HPR0 can mutate to ISAV-HPRΔ.

In Chile, the first ISA outbreak in marine-farmed Atlantic salmon in mid-June 2007 was officially confirmed to be associated with ISAV-HPR7b, which was similar to Norwegian isolates, but had acquired an insertion of 33 base pairs in segment 5 [3]. Phylogenetic analysis of ISAV isolates from different outbreaks suggested that the virus was introduced from Norway in 1996 [3], probably through fertilized salmon eggs [22,23].

Since 2010, ISAV-HPR0 has been detected in Chile in Atlantic salmon at different production stages without any clinical signs of ISA until April 2013, when the re-emerged ISAV-HPRΔ (ISAV-HPR3 and ISAV-HPR14) caused two ISA outbreaks in two marine Atlantic salmon farms [20]. Prior to these outbreaks, the predominant strain was ISAV-HPR7b, which comprised 79% of ISAV isolates from 2007–2010 ISA outbreaks [3]. Phylogenetically, Chilean isolates ISAV-HPR3 and ISAV-HPR14 clustered with ISAV-HPR0, also detected in Chile [20]. ISAV-HPR14 had the motif ^360^PAT^362^ characteristic of Chilean ISAV-HPR0 variant [20]. Subsequently, new ISA outbreaks caused by ISAV-HPR7a and ISAV-HPR7b were recorded in November 2013 and January 2014, respectively. These ISAV isolates are different from the ones previously reported in Virol J 2013, 10:344 [20]. In the present study, we describe the genetic characteristics and relationship between ISAV-HPR7a and ISAV-HPR7b that caused the ISA outbreaks in Chile in 2013 and 2014, and the evolution of the ISAV clades since our original analysis in 2009.

## Results

### General description of the ISA cases

The last ISA outbreak in 2013 was reported in November 2013, in Melinka, XI Region; the first ISA outbreak in 2014 was detected in January 2014, on Chiloe Island, X Region. Table 1 shows general characteristics of the six cases included in this study provided by the affected companies and the National Fisheries Service (Sernapesca, Chile); each case represents a field sample. Table 2 summarizes the analyses of the six cases: three cases (December 2013) were caused by the ISAV-HPR7a strains and three cases (March 2014) were caused by the ISAV-HPR7b strain.

**Table 1:** **General characteristics of six cases included in this study**

**Company**	**Company 1**	**Company 2**
**Case**	**218, 220, 11732**	**272, 302, 304**
Species	Atlantic salmon (*Salmo salar*)	Atlantic salmon (*Salmo salar*)
Geographical area	Melinka	Chiloé
Average weight (g)	3539	1104
Date of first detection	05-12-2013	29-01-2014
Total fish affected	360,129	852,443
Percent weekly mortality	0.0198	0.0071
Percent monthly mortality	0.0642	0.0211
Percent cumulative mortality	0.1792	0.2294
Other diseases	Salmonid rickettsial septicemia (SRS), Heart and skeletal muscle inflammation (HSMI); Caligidosis.	Salmonid rickettsial septicemia (SRS); Caligidosis.

The data was provided by the affected companies and National fisheries Service (SERNAPESCA); case represents a field sample.

SRS: Infection by *Piscirickettsia salmonis.*

HSMI: Associated with infection by piscine orthoreovirus.

Caligidosis: Infection by *Caligus rogercresseyi.*

**Table 2:** **Infectious salmon anemia (ISAV) outbreaks caused by HPR7a and HPR7b viruses in Chile (2013–2014)**

**Analysis/case**	**Case 218**	**Case 220**	**Case 11732**	**Case 302**	**Case 304**	**Case 272**
RT-PCR-ISAV	+	+	+	+	+	+
HPR sequencing	+	+	+	+	+	+
Full sequence segment 5	+	+	+	+	+	+
Full sequence segment 6	+	+	+	+	+	+
Cell culture isolation	-	-	+	-	+	-
HPR type	HPR7a	HPR7a	HPR7a	HPR7b	HPR7b	HPR7b
Genogrup	EU-G3	EU-G3	EU-G3	EU-G3	EU-G3	EU-G3
Segment 5 insert	F-	F-	F+	F-	F-	F-
Virus isolate	CGA/218	CGA/220	CGA/11732	CGA/302	CGA/304	CGA/272
GenBank accession segment 5	-	KJ944289	KJ944293	KJ944295	KJ944297	KJ944291
GenBank accession segment 6	KJ944287	KJ944288	KJ944292	KJ944294	KJ944296	KJ944290

+ Analyzed; − analysis not available; F- without insert; F+ with insert.

### Sequence alignment of ISAV segments 5 and 6 reveals virulence markers of 2013 ISAV-HPR7a and 2014 ISAV-HPR7b strains

Table 3 shows multiple amino acid sequence alignments in the HPR portion of HE obtained from selected ISAV-HPR7 isolates, including those identified in this study (highlighted in bold). ISAV-HPR7a (isolate 220; Chile, 2013) is similar to ISAV-HPR7a from Norway [17], whereas ISAV-HPR7b (isolates 272, 302, and 304; Chile, 2014) is similar to ISAV-HPR7b isolated from Chile in 2007 [3]. The HE proteins of ISAV-HPR7a and ISAV-HPR7b in this study and their closest ISAV relatives have sequence identities from 93.9% to 94.6% (Additional file 1: Table S1).

**Table 3:** **Sequence alignment of the highly polymorphic region (HPR) of the hemagglutinin-esterase (HE) from HPR7 isolates**

**Group**	**ISAV isolate (GenBank Acc. No.)**	**Predicted amino acid sequence**																																									**aa deleted**	**Reference**
HPR7a	Norway H17/96 (AF364891)	T	D	V	K	-	-	-	-	-	-	-	-	-	-	-	-	-	-	-	-	-	-	-	-	-	-	-	T	S	V	L	S	N	T	F	I	S	M	G	V	A	23	Devold *et al*. 2001 [24]
**HPR7a**	**Chile 220 (KJ944288)**	**T**	**D**	**V**	**K**	**-**	**-**	**-**	**-**	**-**	**-**	**-**	**-**	**-**	**-**	**-**	**-**	**-**	**-**	**-**	**-**	**-**	**-**	**-**	**-**	**-**	**-**	**-**	**T**	**S**	**V**	**L**	**S**	**N**	**T**	**F**	**I**	**S**	**M**	**G**	**V**	**A**	**23**	This study
HPR7b	Chile 31991-3 N (FJ786983)	T	D	V	K	-	-	-	-	-	-	-	-	-	-	-	-	-	-	-	-	-	-	-	-	-	-	-	T	S	V	L	S	N	I	F	I	S	M	G	V	A	23	Kibenge *et al.* 2009 [3]
**HPR7b**	**Chile 272 (KJ944290)**	**T**	**D**	**V**	**K**	**-**	**-**	**-**	**-**	**-**	**-**	**-**	**-**	**-**	**-**	**-**	**-**	**-**	**-**	**-**	**-**	**-**	**-**	**-**	**-**	**-**	**-**	**-**	**T**	**S**	**V**	**L**	**S**	**N**	**I**	**F**	**I**	**S**	**M**	**G**	**V**	**A**	**23**	This study
**HPR7b**	**Chile 302 (KJ944294)**	**T**	**D**	**V**	**K**	**-**	**-**	**-**	**-**	**-**	**-**	**-**	**-**	**-**	**-**	**-**	**-**	**-**	**-**	**-**	**-**	**-**	**-**	**-**	**-**	**-**	**-**	**-**	**T**	**S**	**V**	**L**	**S**	**N**	**I**	**F**	**I**	**S**	**M**	**G**	**V**	**A**	**23**	This study
**HPR7b**	**Chile 304 (KJ944296)**	**T**	**D**	**V**	**K**	**-**	**-**	**-**	**-**	**-**	**-**	**-**	**-**	**-**	**-**	**-**	**-**	**-**	**-**	**-**	**-**	**-**	**-**	**-**	**-**	**-**	**-**	**-**	**T**	**S**	**V**	**L**	**S**	**N**	**I**	**F**	**I**	**S**	**M**	**G**	**V**	**A**	**23**	This study
HPR7c	Norway R171/07	T	D	V	K	-	-	-	-	-	-	-	-	-	-	-	-	-	-	-	-	-	-	-	-	-	-	-	T	S	V	L	S	N	I	S	I	S	M	G	V	A	23	Plarre 2011 [25]
HPR7d	-	T	D	V	-	-	R	-	-	-	-	-	-	-	-	-	-	-	-	-	-	-	-	-	-	-	-	-	T	S	V	L	S	N	I	F	I	S	M	G	V	A	23	Plarre 2011 [25]
HPR7e	-	T	D	V	K	-	-	-	-	-	-	-	-	-	-	-	-	-	-	-	-	-	-	-	-	-	-	-	T	S	A	P	S	N	I	F	I	S	M	G	V	A	23	Plarre 2011 [25]
HPR7f	Chile CH03/08	T	D	V	K	-	-	-	-	-	-	-	-	-	-	-	-	-	-	-	-	-	-	-	-	-	-	-	T	S	V	S	S	N	I	S	I	S	M	G	V	A	23	Plarre 2011 [25]
HPR7g	Norway FJ594307	T	D	V	K	-	-	-	-	-	-	-	-	-	-	-	-	-	-	-	-	-	-	-	-	-	-	-	T	S	V	L	S	N	I	F	I	Y	M	G	V	A	23	Plarre 2011 [25]
HPR7h	Chile CH03/08	T	D	V	K	-	-	-	-	-	-	-	-	-	-	-	-	-	-	-	-	-	-	-	-	-	-	-	T	S	V	S	S	N	I	F	I	S	M	G	V	A	23	Plarre 2011 [25]
HPR7i	Norway R171/07	T	D	V	K	-	-	-	-	-	-	-	-	-	-	-	-	-	-	-	-	-	-	-	-	-	-	-	T	S	V	I	S	N	I	S	I	S	M	G	V	A	23	Plarre 2011 [25]

Highlighted in bold were cases analyzed in this study.

Table 4 shows multiple amino acid sequence alignments in the putative proteolytic cleavage site of the F glycoprotein from selected Chilean ISAV-HPR7 isolates, including those identified in this study (highlighted in red). ISAV-HPR7a sequenced directly from fish tissues (CGA/220-5) differed in its segment 5 sequence from the one isolated in cell culture (CGA/11732): the latter had an 18-amino acid insertion between N^265^ and Q^266^ next to the putative F proteolytic cleavage site, LEVVREKGDDTSQSDSFY, which was 100% identical to ISAV RNA segment 1 encoding PB2 polymerase. According to the numbering convention established in previous reports [9,3,10], this new 18-amino acid insertion identified in this study is designated IN5. The ISAV-HPR7b isolate (Chile, 2007) had an 11-amino acid insertion from ISAV RNA segment 2 encoding PB1 polymerase at the putative F proteolytic cleavage site [3]. Thus ISAV-HPR7b isolates from 2014 and 2007 differ in their segment 5 sequences in that the 2014 ISAV-HPR7b had no insertion. The F proteins of ISAV-HPR7a and ISAV-HPR7b in this study and their closest ISAV relatives have sequence identities from 95.4% to 96.8% (Additional file 2: Table S2).

**Table 4:** **Sequence alignment of critical regions of the fusion glycoprotein gene (F) from selected HPR7a isolates**

**Group**	**GenBank Acc. No**	**Predicted amino acid sequence**			**aa inserted**	**Inserts designation**	**Reference**
**HPR7a**	**KJ944293**	**RAGLANQHGWSKYNF- - - - - - - -** **NLEVVREKGDDTSQSDSFYQRA**			**18**	**F+**	**This study**
**HPR7a**	**KJ944289**	**RANLANQHGWSKYSF- - - - - - - -** **N- - - - - - - - - - - - - -** **LRA**			**0**	**F-**	**This study**
**HPR7b**	**KJ944291**	**DAGLANQHGWSKYSF- - - - - - - -N- - - - - - - - - - - - - -LRA**			**0**	**F-**	**This study**
HPR7b	EU849005	RANLANQHGWSKYSF- - - - - - - -NKGKSANDIISD- - - - - -QRA			11	F+	Kibenge et al., 2009 [ 3 ]
HPR7b	EU130923	RANLANQHGWSKYSF- - - - - - - -NKGKSANDIISD- - - - - -QRA			11	F+	Godoy et al., 2008 [ 2 ]

Highlighted in bold were cases analyzed in this study.

### Phylogenetic analyses of ISAV genes encoding HE and F glycoproteins

**Figure 1:**
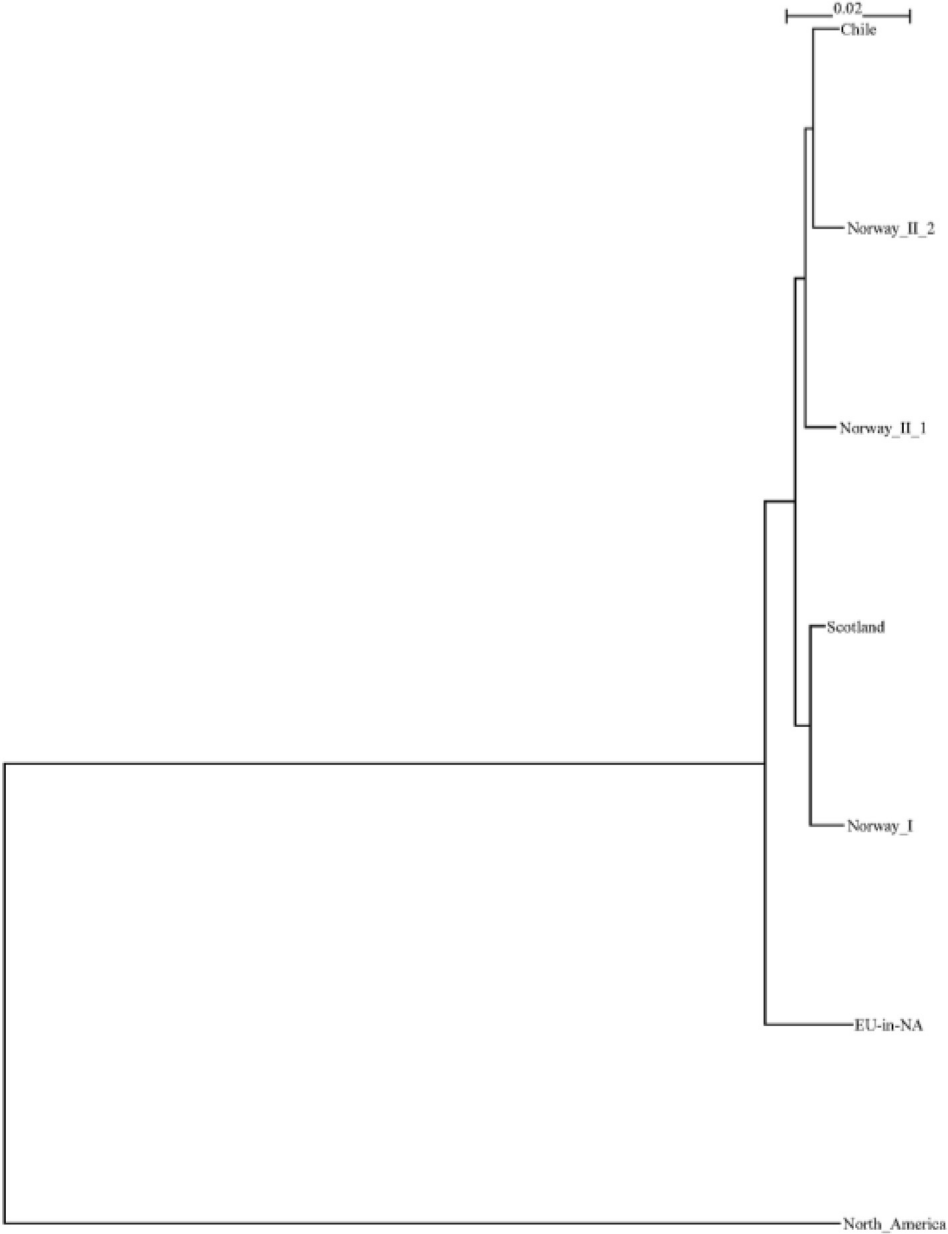
**Phylogenetic tree according to ISAV geographical distribution.** The analysis was performed using 1008 nucleotides of the 5′ portion of the HE gene (excluding the HPR). The phylogenetic tree was constructed by maximum likelihood (ML) using Tamura-Nei and Neighbor-joining.

The phylogenetic analyses of combined segments 5 and 6 sequences for all ISAV isolates for which both segments 5 and 6 sequences are available are presented in Additional file 3: Figure S1. Among the 6 isolates of this study, 5 of them are included in this tree. All together, 151 isolates are included in this tree. By examining Additional file 3: Figure S1 and using our previously reported data [3], we chose one representative isolate from every geographical region and constructed a phylogenetic tree that revealed the relationship between the different geographical isolates and possible transmission routes among the regions. This tree is presented in Figure 1. The following ISAV isolates were chosen: 98-049-1 represents North America; 04-085-1 represents EU-in-NA; 810/9/99 represents Norway I; 93/09/2264 represents Norway_II_1; SK779/06 represents Norway_II_2; U24636 represents Chile; and 390/98 represents Scotland (Figure 1).

Figure 1 summarizes major conclusions of ISAV research. In this study, we concentrated on Chilean ISAV isolates which are most likely evolved from Norway_II, including Norway_II_1 and Norway_II_2. This notion was first proposed by Kibenge *et al.* [3] and was confirmed by this and other studies.

**Figure 2:**
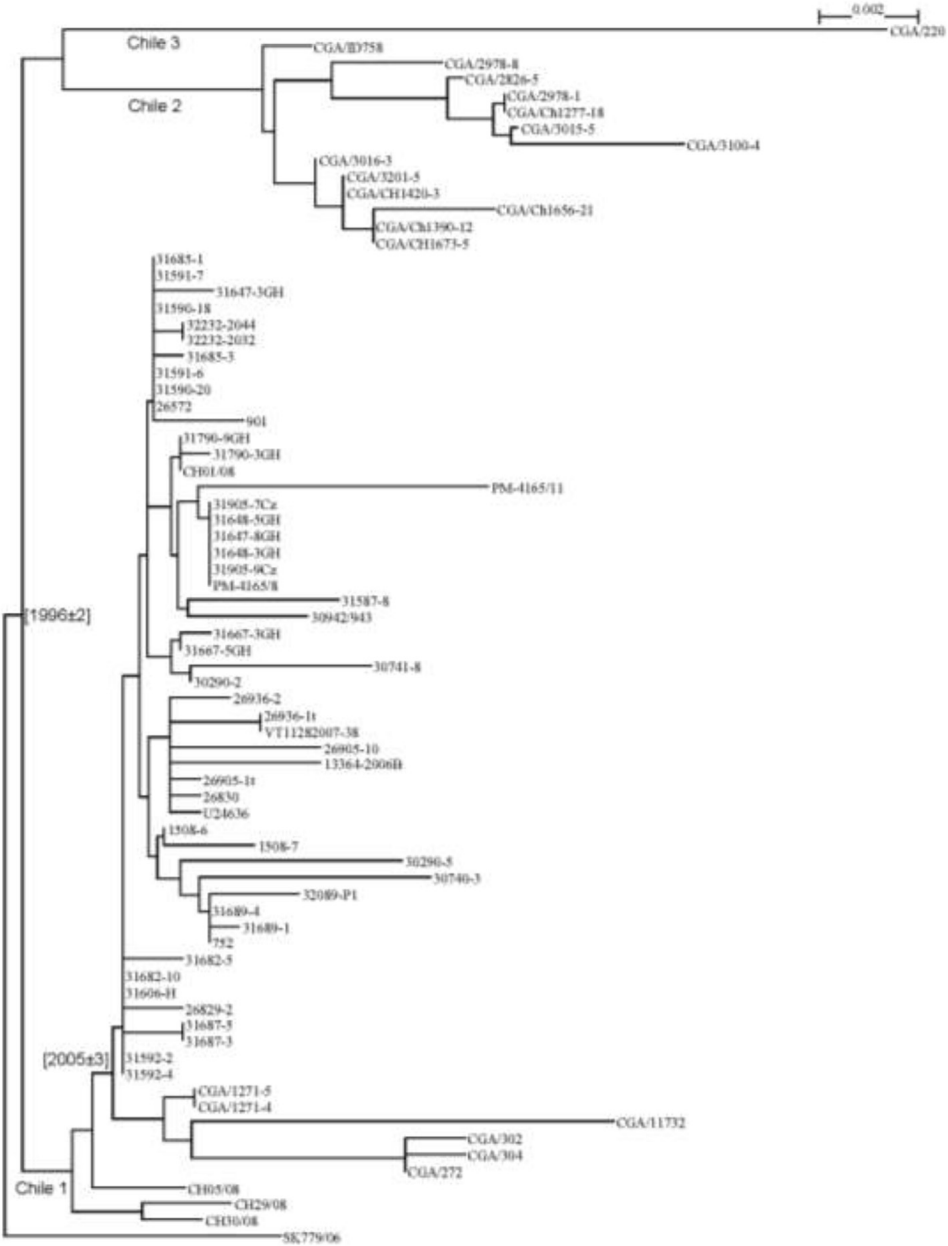
**Phylogenetic tree of 74 Chilean ISAV isolates based on segment 5 and 6 sequences.** The tree indicates that all Chilean ISAV isolates can be divided into three major groups: Chile 1, Chile 2, and Chile 3.

In view of these data, we performed phylogenetic analysis of Chilean ISAV isolates using a typical Norway_II_2 isolate as outgroup. A phylogenetic tree comprising 74 Chilean ISAV isolates for which segment 5 and 6 sequences are available, and one outgroup is presented in Figure 2. The tree shows that all Chilean ISAV isolates can be divided into three major groups: Chile 1 is the biggest group including 60 previous isolates [3]; among the five isolates from the present study, four (272, 302, 304, 11732) belong to this group. The group Chile 2 contains 13 isolates from 2013; most of them have been discussed [20]. The group Chile 3 consists of only one isolate, CGA/220, which has its characteristic features and could therefore be recognized as a new clade within Chilean ISAV isolates. It is expected that future work will support this discovery.

The time of genetic divergence at key branching points have been reported by Kibenge *et al.* [3] before Chile 2 and 3 were collected and three 2008 isolates (CH05/08, CH29/08, and CH30/08) were not included. The two time points related to this study are marked in Figure 2; the main timeline is still consistent with Kibenge et al. ([3], Figure six). We believe that the nomenclature of ISAV isolates presented in Figure 1 is stable and the genotypes, genogroups, and major clades described by Kibenge *et al.* [3] are mature. Referring to the clades within Chilean isolate group which is still in the active stage of evolution, we may use Chile 1, 2, and 3 until further information is available to propose formal nomenclature.

### Phylogenetic analyses of the ISAV genes encoding HE and F glycoproteins reveals clustering of Chile isolates suggestive of different transmission pathways

**Figure 3:**
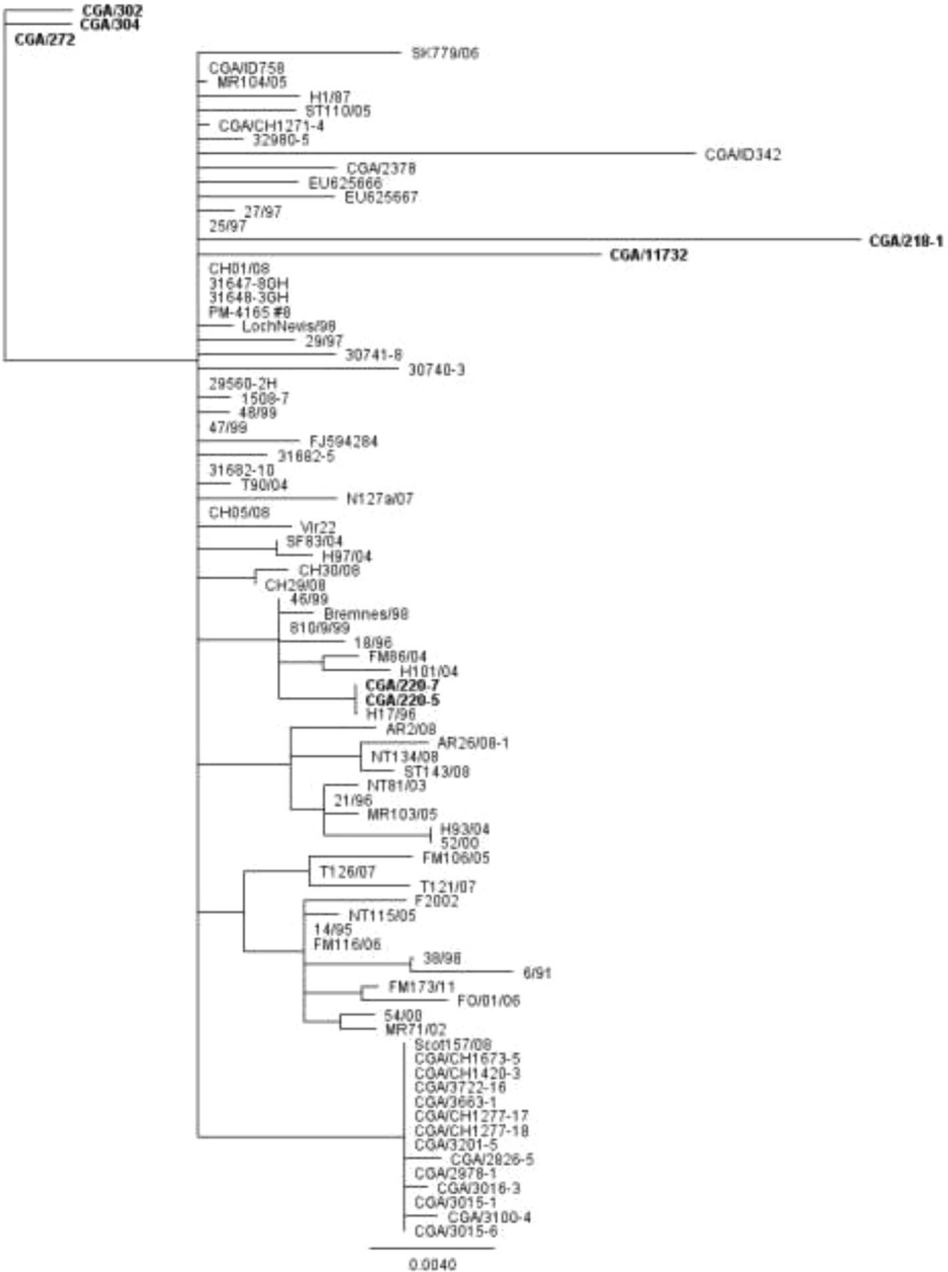
**Phylogenetic tree of segment 6 sequences from selected ISAV isolates of European genotype.** The analysis was performed using 1008 nucleotides of the 5′ portion of the HE gene (excluding the HPR). The phylogenetic tree was constructed by maximum likelihood (ML) using Tamura-Nei and Neighbor-joining. ISAV-HPR7a and HPR7b associated with the 2014 ISA outbreaks in Chile are shown in bold.

**Figure 4:**
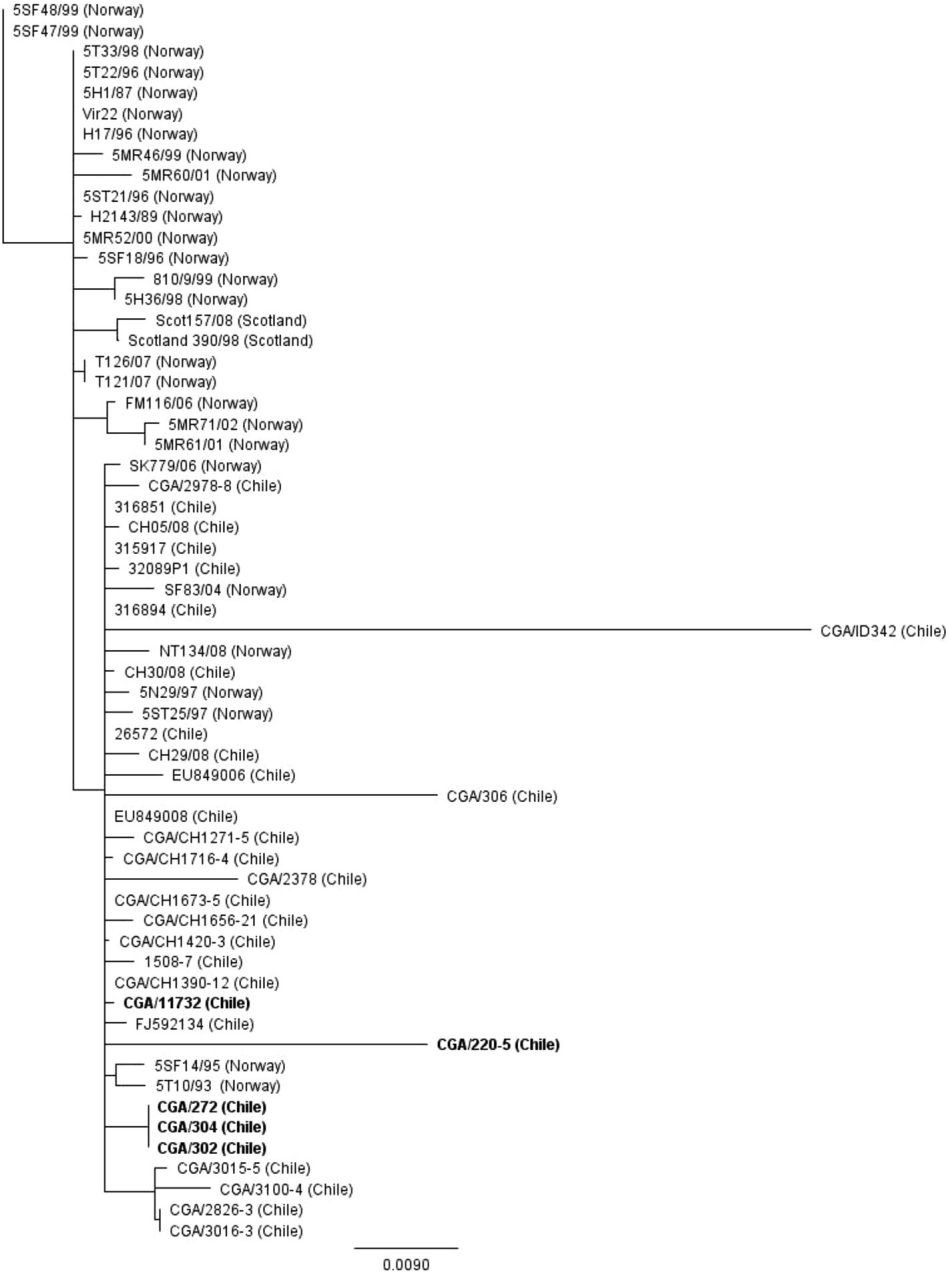
**Phylogenetic tree of segment 5 sequences from selected ISAV isolates of European genotype.** The analysis was performed using 808 nucleotides of the 5′ portion of the F gene (excluding the nucleotides responsible for Q^266^ → L^266^ substitution). The phylogenetic tree was constructed by maximum likelihood using the neighbor-joining method and Tamura-Nei genetic distances. All ISAV-HPR7a and HPR7b viruses associated with the 2014 ISA outbreaks in Chile are shown in bold.

The ISAV isolates, for which the sequences for segment 5 or 6 were not available, were excluded from the phylogenetic analysis. However, such analysis for each segment would be more comprehensive and may reveal segment-specific mutations. The phylogenetic analysis of segment 6 and 5 sequences from selected ISAV isolates of European lineage (genogroup EU-G3) [4], including 2013 ISAV-HPR7a with (CGA/218-1, CGA/11732) and without (CGA/220-5, CGA/220-7) insert sequence in segment 5, and from 2014 ISAV-HPR7b of this study are presented in Figures 3 and 4, respectively. According to segment 6 sequence, CGA/220-5 and CGA/220-7 cluster with ISAV H17/96 from Norway [19], whereas CGA/218-1 and CGA/11732 are close to Norwegian isolates ISAV 27/97, 25/97 and ISAV 29/97, and 2007 ISAV-HPR7b. The 2014 ISAV-HPR7b isolates (CGA/272, CGA/320, and CGA/304) cluster separately with ISAV Vir 22 from Norway [10] and with ISAV-HPR3 and ISAV-HPR14 responsible for the April 2013 ISA outbreaks in Chile (Figure 3).

The phylogenetic analysis of segment 5 shows that both 2013 ISAV-HPR7a isolates cluster together with Norwegian isolates ISAV 25/97 and ISAV 29/97, and 2007 ISAV-HPR7b, whereas the 2014 ISAV-HPR7b isolates (CGA/272, CGA/320, and CGA/304) remain clustered with ISAV-HPR3 and ISAV-HPR14 (Figure 4), which is consistent with the segment 6-based grouping.

## Discussion

The last ISA outbreak in 2013 in Chile was attributed to ISAV-HPR7a; this virus did not have a sequence insertion in segment 5 when sequenced directly from salmon tissues, but had this insertion when the virus isolated from cell culture was sequenced. The first ISA outbreak reported in 2014 in Chile was associated with ISAV-HPR7b without insertion in segment 5. Both viruses had a 23-amino acid deletion in HPR of segment 6, but differed in that HPR7a had threonine and HPR7b had isoleucine at position 370. In segment 5, the salmon tissue-originated ISAV-HPR7a had Q^266^ → L^266^ substitution, whereas the cell culture-grown ISAV-HPR7a had an 18-amino acid insertion LEVVREKGDDTSQSDSFY of 100% sequence identity with ISAV RNA segment 1 encoding PB2 polymerase. These data indicate the variability in the two ISAV virulence markers, especially in case of the ISAV-HPR7a isolate, depending on virus growth conditions (salmon tissue or cell culture). The virulent ISAV-HPRΔ strains have deletion in HPR as well as Q^266^ → L^266^ substitution or insertion in the putative proteolytic site of the F protein [10]. The last ISA outbreak of 2010 was caused by ISAV-HPR7b strains with and without insertion in segment 5, but none of them was similar to the ISAV-HPR7b isolate responsible for ISA outbreak in 2007, which had an 11-amino acid insertion of 100% sequence identity with ISAV RNA segment 2 in the F protein [3]. This insertion might have occurred through non-homologous recombination between the F- and PB1-encoding genes of the same virus; more than 85% of the outbreak cases were caused by this virus [3]. The last ISA outbreaks in Chile confirm the variability in ISAV virulence markers because the 2013 cases were associated with ISAV-HPR3 and ISAV-HPR14 without sequence insertions in segment 5 [20], supporting the quasispecies hypothesis, which suggests that multiple evolutionary forces account for ISAV genetic diversity [3]. The ISAV virulence in the six cases in this study was different too: ISAV-HPR7a appeared to be more virulent than ISAV-HPR7b without sequence insertion in segment 5 (MGG, personal communication). It is therefore possible that the sequence insertion in segment 5 of the 2007 ISAV-HPR7b isolate contributed to the virulence of the virus.

The virulent ISAV isolates in Chile show a close relationship with ISAV-HPR0 [20]; by 2012, the low-pathogenic ISAV-HPR0 strain had completely replaced the virulent ISAV-HPR∆ strain responsible for the 2007–2010 ISA outbreaks, as the dominant ISAV variant in marine-farmed Atlantic salmon in Chile. The occurrence of two new ISA outbreaks in April 2013 marked a brief re-emergence of virulent ISAV-HPR∆, demonstrating that ISAV-HPR0 dissemination together with the sporadic ISAV-HPR∆ returns is a characteristic feature of ISA post-crisis dynamics [12,14,16,17].

Based on our understanding about virus transmissions where a virus transmission occurrence can be roughly classified into the latent stage and the breakout stage; and mutation rates tend to be slow in the latent stage and fast in the breakout stage, the variable mutation rate model used in Figure 2 is consistent with a typical virus transmission. Typically, a small amount of virus is physically transmitted to a new location and finds suitable hosts and the latent stage starts. In this stage, mutation occurs at a slow rate and in a unified way. When the amount of virus accumulates to a certain level, the breakout stage starts and the mutation rate is faster. ISAV isolates from Norway transmitted to Chile around 1996 might have existed in the same or different locations in Chile and gone through different evolution pathways: Chile 1 entered the breakout stage early around 2005; Chile 2 entered the breakout stage at some time before 2013; and Chile 3 might still be in the latent stage. The phylogenetic tree of the concatenated segment 5 and 6 sequence (Figure 2) confirms that the 1996 divergence point is still evident in the Chilean ISAV isolates.

## Conclusion

In summary, in the last two to three years, we observed active evolution of Chilean ISAV isolates. The samples collected in 2013 and 2014 indicate that ISAV has evolved to an extent that one new group (Chile 2) has already been confirmed and another new group (Chile 3) has emerged and will probably be recognized as such in the near future.

## Materials and methods

### Study material

The study material included samples from six ISA cases in Chile: three cases detected in December 2013 were caused by ISAV-HPR7a strain and three cases in March 2014 were associated with ISAV-HPR7b strain. Necropsy was performed on Atlantic salmon moribund fish reared in seawater cages and the main clinical signs were recorded. Samples were collected and submitted for virus isolation and detection by reverse transcription quantitative polymerase chain reaction (RT-qPCR) to the ETECMA Laboratory (Puerto Montt, Chile); nucleotide sequencing was performed in the Centro de Investigaciones Biologicas Aplicadas (CIBA; Puerto Montt, Chile).

### Virus isolation

Tissue samples for virus isolation were collected in Mínimum Essential Media (GIBCO®, Life Technologies) and were shipped on ice to the laboratory. Homogenized heart, spleen, and kidney tissues were used to infect monolayers of Chinook salmon embryo (CHSE-214), Atlantic salmon Kidney (ASK-1), Atlantic salmon head kidney (SHK-1), and Bluegill Fry (BF-2) cell lines following the standard protocols in the OIE Aquatic Manual [26].

For virus propagation, cells were seeded in tissue culture flasks for 24 h; medium was removed and virus-containing supernatants diluted 1:10 in serum-free medium were added for 2 h at room temperature to allow virus adsorption. Growth medium was then added and the inoculated cells were incubated at 16°C; infection was monitored daily using an inverted light microscope until cytopathic effect (CPE) was evident or up to 21 days and the flasks were frozen at −80°C. Virus replication was also monitored by RT-PCR in cell lysates since it may occur without apparent CPE [27]. The cultures negative by CPE and RT-PCR were passaged on fresh cell monolayers. A sample was considered negative if no CPE or RT-PCR amplification was observed after three blind passages.

### Total RNA extraction

Automated tissue homogenization was performed using the MagNA Lyser**®** instrument (Roche). For RNA isolation, a Roche MagNA Pure LC**®** (Roche) instrument was used. Total viral RNA was extracted using the MagNA Pure LC RNA isolation kit III-Tissue**® **(Roche) and bacterial and fungal DNA was isolated using the MagNA Pure LC DNA Isolation kit III–Tissue**® **(Roche)**,** according to the manufacturer’s instructions.

### Real-time RT-PCR

Primers specific for ISAV segment 8 and PCR conditions were described by Snow *et al.* [28]. Samples were considered ISAV-positive based on cycle threshold (Ct) values <30 according to the laboratory procedure. The confirmation of ISAV-HPR-positive cases was performed by sequencing RT-PCR products obtained using segment 6 HPR primers as previously described [3,12,17] and/or by specific detection of ISAV-HPR by RT-qPCR using TaqMan**®** system [20]. To ensure efficient performance of each assay, a constitutively expressed eukaryotic elongation factor 1-alpha (ELF1α) was used as an internal control reference gene [28]. RT-qPCR was conducted using LightCycler 480 RNA Master Hydrolysis Probes**®** for RNA and LightCycler 480 Probe Master**®** (Roche).

### Nucleotide sequencing and sequence analysis

HPR segment 6 of all ISAV-HPRΔ-positive samples was amplified using the primer set reported by Kibenge *et al.* [3]. Full-length segments 5 and 6 were amplified using the primers published by McBeath *et al.* [15]. Conventional RT-PCR was carried out using the EXPRESS qPCR SuperMix**®** with Premixed ROX**®** (Invitrogen) in a Swift™ MaxPro Thermal Cycler**®** (Esco Healthcare Pte. Ltd.). PCR products were purified by agarose gel electrophoresis and directly sequenced. RNA samples were submitted to the Virology Research Laboratory (University Prince Edward Island, Canada) for confirmation of the sequencing results. The sequences were analyzed with Geneious v 6.0.6 software and subjected to a BLAST search against the latest release at GenBank. Sequences of segments 6 and 5 from the six cases in this study (Table 1) and have been deposited in the GenBank database under accession numbers listed in Table 2. Additional GenBank Accession numbers used in the phylogenetic analyses and the multiple alignments are shown in Additional file 4: Table S3.

The evolutionary relationship of ISAV-HPRΔ HE gene sequenced in this study and the reference sequences retrieved from GenBank was determined using the neighbor-joining method [29]. A bootstrap analysis to investigate the stability of phylogenetic trees was performed on 1,000 replicates. Full-length segments 5 and 6 were processed into two corresponding sets of multiple sequence alignment files (ISAV-HPRΔ F and ISAV-HPRΔ HE gene sequences).

Combined segments 5 and 6 sequences were obtained directly from GenBank [30] and checked to ensure that they were updated, unique, and correct. CLUSTAL *X*2 with the default settings was used for multiple sequence alignment of each viral RNA segment; the same reference isolates were then used for phylogenetic analysis. For RNA segment 6, only the first 1,008 nucleotides were used. Bootstrap analysis to investigate the stability of phylogenetic trees was performed on 1,000 replicates.

## Additional files

Additional file 1: Table S1. The percentages of sequence identity of the viral gene encoding hemagglutinin-esterase (segment 6) of ISAV-HPR7a and ISAV-HPR7b isolated in this study (red) and their closest ISAV relatives. Description: Table summarizing the sequence identity on segment 6.) Additional file 2: Table S2. The percentages of sequence identity of the viral gene encoding the Fusion protein (segment 5) of ISAV-HPR7a and ISAV-HPR7b isolated in this study (red) and their closest ISAV relatives. Description: Table summarizing the sequence identity on segment 5. Additional file 3: Figure S1. Phylogenetic tree of combined RNA segments 5 and 6 showing the relationships between all ISAV isolates. Description: The analysis was performed for both segments (excluding HPR). The phylogenetic tree was constructed by maximum likelihood using the neighbor-joining method and Tamura-Nei genetic distances. Additional file 4: Table S3. Additional GenBank Accession numbers used in the phylogenetic analyses and the multiple alignments.
