# Supplementary material for: Genetic analysis and comparative virulence of infectious salmon anemia virus (ISAV) types HPR7a and HPR7b from recent field outbreaks in Chile
Source: Virol J. 2014 Nov 29;11:204. doi: 10.1186/s12985-014-0204-1 (PMC4272776; doi:10.1186/s12985-014-0204-1)
Supplement: Additional file 1: Table S1. — The percentages of sequence identity of the viral gene encoding hemagglutinin-esterase (segment 6) of ISAV-HPR7a and ISAV-HPR7b isolated in this study (red) and their closest ISAV relatives. Description: Table summarizing the sequence identity on segment 6.) [file 12985_2014_204_MOESM1_ESM.doc]

|  | CGA/3722-16 | CGA/CH1420-3 | CGA/CH1673-5 | Scot157/08 | CGA/2826-5 | 25/97 | **CGA/218-1** | **CGA/220-5** | **CGA/220-7** | **CGA/11732** | H17/96 | 29/97 | 29560-2H | **CGA/272** | **CGA/320** | **CGA/304** | 46/99 | 810/9/99 | Bremnes/98 | HPR19_18/96 |
| --- | --- | --- | --- | --- | --- | --- | --- | --- | --- | --- | --- | --- | --- | --- | --- | --- | --- | --- | --- | --- |
| CGA/3722-16 | 100 | 99.2 | 99.2 | 99.2 | 99 | 98.1 | **95.1** | **95.3** | **95.3** | **97.8** | 97.7 | 97.9 | 97.8 | **97.4** | **97.1** | **97.1** | 98 | 97.9 | 97.8 | 97.8 |
| CGA/CH1420-3 |  |  | 99.9 | 99.9 | 99.8 | 98.8 | **95.7** | **95.9** | **95.9** | **98.6** | 98.3 | 98.6 | 98.5 | **98.1** | **97.9** | **97.9** | 98.7 | 98.6 | 98.5 | 98.5 |
| CGA/CH1673-5 |  |  |  | 100 | 99.7 | 98.9 | **95.6** | **96** | **96** | **98.5** | 98.5 | 98.7 | 98.6 | **98** | **97.8** | **97.8** | 98.8 | 98.7 | 98.6 | 98.6 |
| Scot157/08 |  |  |  |  | 99.7 | 98.9 | **95.6** | **96** | **96** | **98.5** | 98.5 | 98.7 | 98.6 | **98** | **97.8** | **97.8** | 98.8 | 98.7 | 98.6 | 98.6 |
| CGA/2826-5 |  |  |  |  |  | 98.8 | **95.9** | **95.9** | **95.9** | **98.8** | 98.3 | 98.6 | 98.7 | **98.3** | **98.1** | **98.1** | 98.7 | 98.6 | 98.5 | 98.5 |
| 25/97 |  |  |  |  |  |  | **96.6** | **96.7** | **96.7** | **99.3** | 99.1 | 99.3 | 99.7 | **98.9** | **98.7** | **98.7** | 99.4 | 99.3 | 99.2 | 99.2 |
| **CGA/218-1** |  |  |  |  |  |  |  | **93.7** | **93.7** | **96.8** | **96** | **96.3** | **96.7** | **96.3** | **96** | **96** | **96.4** | **96.3** | **96.1** | **96.1** |
| **CGA/220-5** |  |  |  |  |  |  |  |  | **100** | **96.3** | **97.6** | **96.5** | **96.4** | **95.9** | **95.7** | **95.7** | **97.2** | **97.1** | **97** | **97** |
| **CGA/220-7** |  |  |  |  |  |  |  |  |  | **96.3** | **97.6** | **96.5** | **96.4** | **95.9** | **95.7** | **95.7** | **97.2** | **97.1** | **97** | **97** |
| **CGA/11732** |  |  |  |  |  |  |  |  |  |  | **98.7** | **99.1** | **99.4** | **99.1** | **98.9** | **98.9** | **99** | **98.9** | **98.8** | **98.8** |
| H17/96 |  |  |  |  |  |  |  |  |  |  |  | 98.9 | 98.8 | **98.2** | **98** | **98** | 99.7 | 99.6 | 99.4 | 99.4 |
| 29/97 |  |  |  |  |  |  |  |  |  |  |  |  | 99.2 | **98.8** | **98.6** | **98.6** | 99.2 | 99.1 | 99 | 99 |
| 29560-2H |  |  |  |  |  |  |  |  |  |  |  |  |  | **99** | **98.8** | **98.8** | 99.1 | 99 | 98.9 | 98.9 |
| **CGA/272** |  |  |  |  |  |  |  |  |  |  |  |  |  |  | **99.8** | **99.8** | **98.6** | **98.5** | **98.3** | **98.3** |
| **CGA/320** |  |  |  |  |  |  |  |  |  |  |  |  |  |  |  | **99.6** | **98.3** | **98.2** | **98.1** | **98.1** |
| **CGA/304** |  |  |  |  |  |  |  |  |  |  |  |  |  |  |  |  | **98.3** | **98.2** | **98.1** | **98.1** |
| 46/99 |  |  |  |  |  |  |  |  |  |  |  |  |  |  |  |  |  | 99.9 | 99.8 | 99.8 |
| 810/9/99 |  |  |  |  |  |  |  |  |  |  |  |  |  |  |  |  |  |  | 99.9 | 99.7 |
| Bremnes/98 |  |  |  |  |  |  |  |  |  |  |  |  |  |  |  |  |  |  |  | 99.6 |
| 18/96 |  |  |  |  |  |  |  |  |  |  |  |  |  |  |  |  |  |  |  | 100 |
