# Supplementary material for: Genetic analysis and comparative virulence of infectious salmon anemia virus (ISAV) types HPR7a and HPR7b from recent field outbreaks in Chile
Source: Virol J. 2014 Nov 29;11:204. doi: 10.1186/s12985-014-0204-1 (PMC4272776; doi:10.1186/s12985-014-0204-1)
Supplement: Additional file 2: Table S2. — The percentages of sequence identity of the viral gene encoding the Fusion protein (segment 5) of ISAV-HPR7a and ISAV-HPR7b isolated in this study (red) and their closest ISAV relatives. Description: Table summarizing the sequence identity on segment 5. [file 12985_2014_204_MOESM2_ESM.doc]

|  | 5H1/87 | 5T22/96 | 5T33/98 | CGA/2826-3 | CGA/3016-3 | 5T10/93 | 5SF14/95 | **CGA/11732** | H17/96 | 5N29/97 | 26572 | **CGA/272** | **CGA/302** | **CGA/304** | **CGA/306** | EU849006 | 1508-7 | CGA/3015-5 | CGA/3100-4 |
| --- | --- | --- | --- | --- | --- | --- | --- | --- | --- | --- | --- | --- | --- | --- | --- | --- | --- | --- | --- |
| 5H1/87 | 100 | 99.9 | 99.9 | 99.1 | 99.1 | 98.8 | 98.8 | **98.5** | 100 | 98.3 | 98.6 | **98.4** | **98.4** | **98.4** | **94.7** | 98.3 | 98.5 | 98.9 | 98.5 |
| 5T22/96 |  |  | 100 | 99 | 99 | 98.6 | 98.6 | **98.4** | 99.9 | 98.1 | 98.5 | **98.3** | **98.3** | **98.3** | **94.6** | 98.1 | 98.4 | 98.8 | 98.4 |
| 5T33/98 |  |  |  | 99 | 99 | 98.6 | 98.6 | **98.4** | 99.9 | 98.1 | 98.5 | **98.3** | **98.3** | **98.3** | **94.6** | 98.1 | 98.4 | 98.8 | 98.4 |
| CGA/2826-3 |  |  |  |  | 100 | 98.4 | 98.4 | **98.6** | 99.1 | 97.9 | 98.8 | **98.5** | **98.5** | **98.5** | **94.7** | 98.4 | 98.6 | 99.8 | 99.1 |
| CGA/2978-8 |  |  |  |  | 99 | 98.6 | 98.6 | **99.1** | 98.9 | 98.1 | 99 | **99** | **99** | **99** | **95** | 98.6 | 98.9 | 98.8 | 98.6 |
| CGA/3016-3 |  |  |  |  |  | 98.4 | 98.4 | **98.6** | 99.1 | 97.9 | 98.8 | **98.5** | **98.5** | **98.5** | **94.7** | 98.4 | 98.6 | 99.8 | 99.1 |
| 5T10/93 |  |  |  |  |  |  | 99.5 | **98.8** | 98.8 | 99 | 99.1 | **98.6** | **98.6** | **98.6** | **94.9** | 98.5 | 98.8 | 98.4 | 97.5 |
| 5SF14/95 |  |  |  |  |  |  |  | **98.8** | 98.8 | 99 | 99.1 | **98.6** | **98.6** | **98.6** | **94.9** | 98.5 | 98.8 | 98.4 | 97.5 |
| **CGA/11732** |  |  |  |  |  |  |  |  | **98.5** | **98.6** | **99.6** | **99.4** | **99.4** | **99.4** | **95.4** | **99** | **99.3** | **98.4** | **97.8** |
| H17/96 |  |  |  |  |  |  |  |  |  | 98.3 | 98.6 | **98.4** | **98.4** | **98.4** | **94.7** | 98.3 | 98.5 | 98.9 | 98.5 |
| 5N29/97 |  |  |  |  |  |  |  |  |  |  | 99 | **98.4** | **98.4** | **98.4** | **94.7** | 98.5 | 98.6 | 97.9 | 97 |
| 26572 |  |  |  |  |  |  |  |  |  |  |  | **99.3** | **99.3** | **99.3** | **95.5** | 99.4 | 99.6 | 98.8 | 97.9 |
| **CGA/272** |  |  |  |  |  |  |  |  |  |  |  |  | **100** | **100** | **95.3** | **98.6** | **98.9** | **98.3** | **97.6** |
| **CGA/302** |  |  |  |  |  |  |  |  |  |  |  |  |  | **100** | **95.3** | **98.6** | **98.9** | **98.3** | **97.6** |
| **CGA/304** |  |  |  |  |  |  |  |  |  |  |  |  |  |  | **95.3** | **98.6** | **98.9** | **98.3** | **97.6** |
| **CGA/306** |  |  |  |  |  |  |  |  |  |  |  |  |  |  |  | **94.9** | **95.2** | **94.6** | **94.1** |
| EU849006 |  |  |  |  |  |  |  |  |  |  |  |  |  |  |  |  | 99.3 | 98.4 | 97.5 |
| 1508-7 |  |  |  |  |  |  |  |  |  |  |  |  |  |  |  |  |  | 98.6 | 97.8 |
| CGA/3015-5 |  |  |  |  |  |  |  |  |  |  |  |  |  |  |  |  |  |  | 99.1 |
| CGA/3100-4 |  |  |  |  |  |  |  |  |  |  |  |  |  |  |  |  |  |  | 100 |
