# Supplementary figures and images for: Genetic analysis and comparative virulence of infectious salmon anemia virus (ISAV) types HPR7a and HPR7b from recent field outbreaks in Chile
Source: Virol J. 2014 Nov 29;11:204. doi: 10.1186/s12985-014-0204-1 (PMC4272776; doi:10.1186/s12985-014-0204-1)

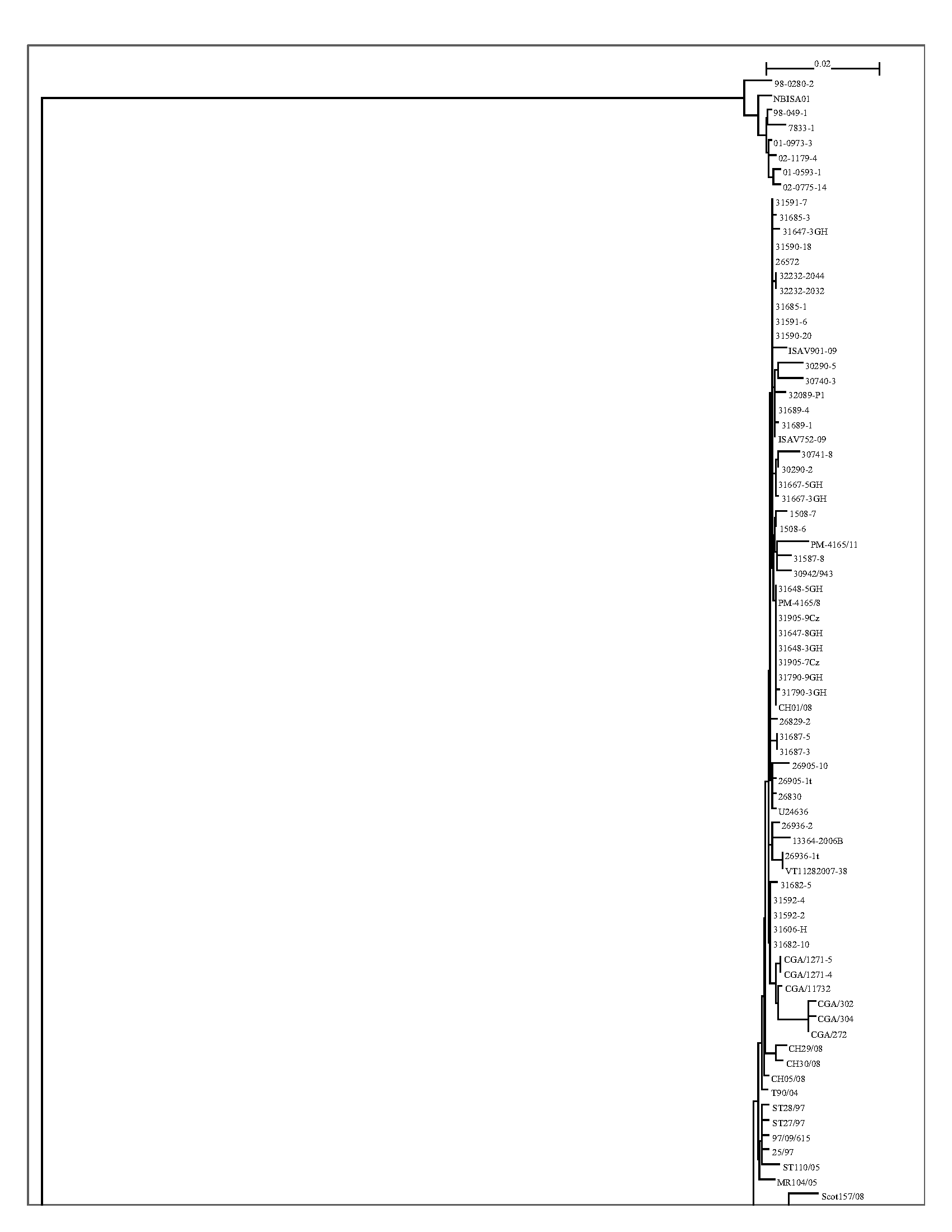


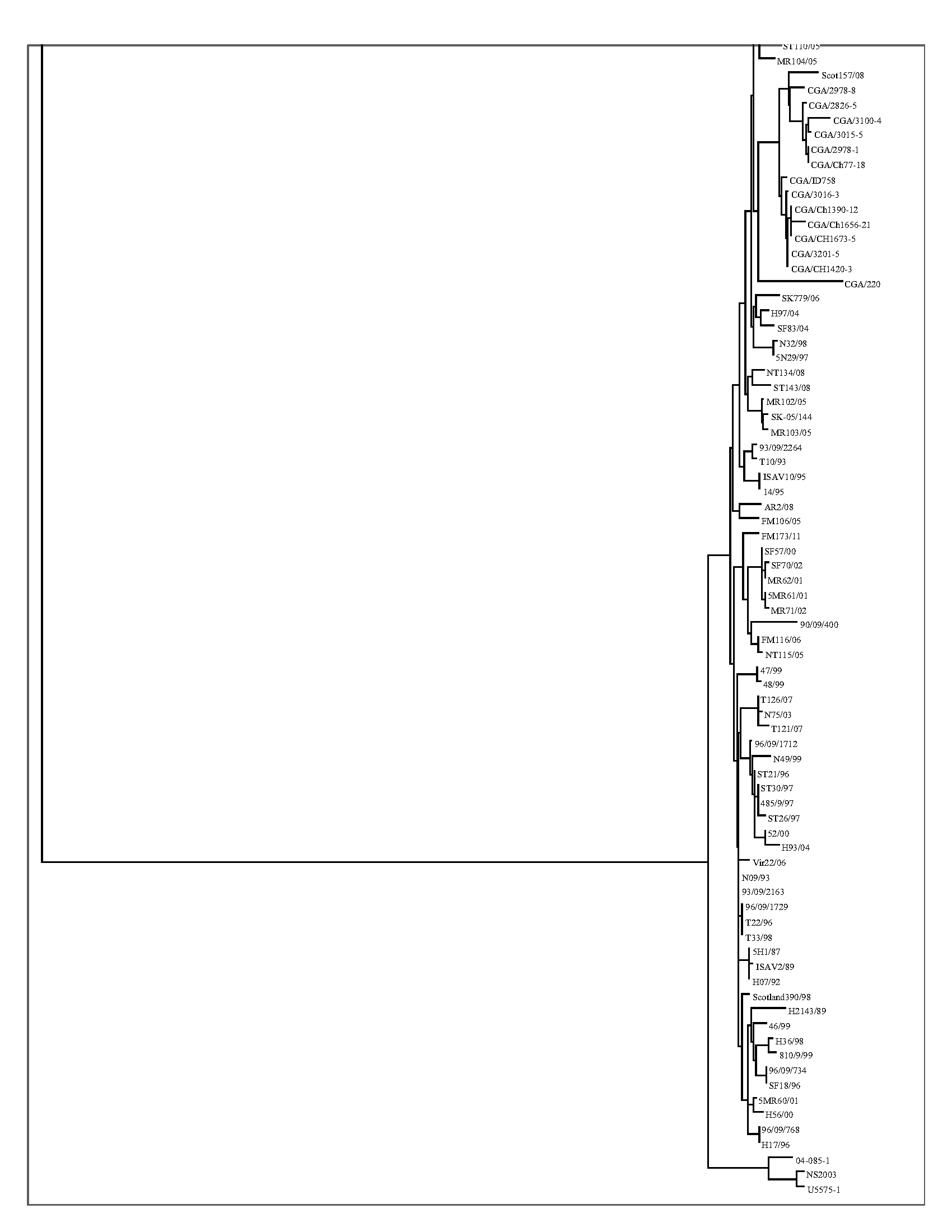

Supplement: Additional file 3: Figure S1. — Phylogenetic tree of combined RNA segments 5 and 6 showing the relationships between all ISAV isolates. Description: The analysis was performed for both segments (excluding HPR). The phylogenetic tree was constructed by maximum likelihood using the neighbor-joining method and Tamura-Nei genetic distances. [file 12985_2014_204_MOESM3_ESM.doc]
