# Supplementary material for: Genetic analysis and comparative virulence of infectious salmon anemia virus (ISAV) types HPR7a and HPR7b from recent field outbreaks in Chile
Source: Virol J. 2014 Nov 29;11:204. doi: 10.1186/s12985-014-0204-1 (PMC4272776; doi:10.1186/s12985-014-0204-1)
Supplement: Additional file 4: Table S3. — Additional GenBank Accession numbers used in the phylogenetic analyses and the multiple alignments. [file 12985_2014_204_MOESM4_ESM.doc]

**Supplementary Table 3.** Additional GenBank Accession numbers used in the phylogenetic analyses and the multiple alignments.

| **HPR type** | **Isolate** | **Country** | **Segment 5** | **Segment 6** | **Reference** | **Year** |
| --- | --- | --- | --- | --- | --- | --- |
| HPR0 | AR2/08 | Norway | FN687418 | FN687348 | Lyngstad *et al.* [17] | 2008 |
| HPR0 | AR26/08-1 | Norway | - | FN687356 | Lyngstad *et al.* [17] | 2008 |
| HPR0 | FM173/11 | Norway | JN711019 | JN711060 | Plarre [25] | 2011 |
| HPR0 | FO/01/06 | Faroe Island | - | HQ664992 | Christiansen *et al.* [16] | 2006 |
| HPR0 | H97/04 | Norway | JN711024 | DQ108604 | Nylund *et al.* [4] | 2004 |
| HPR0 | MR104/05 | Norway | JN711026 | DQ108607 | Nylund *et al.* [4] | 2005 |
| HPR0 | Scot157/08 | Scotland | JN711010 | JN711096 | Plarre *et al.* [19] | 2008 |
| HPR0 | SF83/04 | Norway | AY744392 | AY973190 | Nylund *et al.* [4] | 2004 |
| HPR0 | SK779/06 | Norway | EU118819 | EU118820 | Markussen *et al.* [10] | 2006 |
| HPR0 | CH29/08 | Chile | JN711012 | JN711094 | Plarre [25] | 2008 |
| HPR0 | CH30/08 | Chile | JN711013 | JN711095 | Plarre [25] | 2008 |
| HPR0 | CGA/CH1656-21 | Chile | KF413748 | KF413749 | Godoy *et al.* [20] | 2013 |
| HPR0 | CGA/CH1390-12 | Chile | KF373252 | KF373253 | Godoy *et al.* [20] | 2013 |
| HPR0 | CGA/CH1420-3 | Chile | KF373254 | KF373255 | Godoy *et al.* [20] | 2013 |
| HPR0 | CGA/CH1673-5 | Chile | KF413750 | KF413751 | Godoy *et al.* [20] | 2013 |
| HPR0 | CGA/ID758 | Chile | KF019741 | KF019742 | Godoy *et al.* [20] | 2013 |
| HPR1 | FM86/04 | Norway | - | AY971659 | Devold *et al.* [9] | 2004 |
| HPR1 | 5H1/87 | Norway | AY853942 | AF364893 | Devold *et al.* [24] | 1987 |
| HPR2 | 6/91 | Norway | - | AF364894 | Devold *et al.* [9] | 1991 |
| HPR2 | 38/98 | Norway | - | AF364874 | Devold *et al.* [24] | 1998 |
| HPR2 | 5T22/96 | Norway | AY853968 | - | Devold *et al.* [9] | 1996 |
| HPR2 | 5T33/98 | Norway | AY853969 | - | Devold *et al.* [9] | 1998 |
| HPR3 | CGA/2826-5 | Chile | KF051913 | KF051871 | Godoy *et al.* [20] | 2013 |
| HPR3 | CGA/2978-1 | Chile | KF051927 | KF051882 | Godoy *et al.* [20] | 2013 |
| HPR3 | CGA/3016-3 | Chile | KF373256 | KF373257 | Godoy *et al.* [20] | 2013 |
| HPR3 | CGA/3201-5 | Chile | KF373258 | KF373259 | Godoy *et al.* [20] | 2013 |
| HPR3 | CGA/3663-1 | Chile | - | KF373260 | Godoy *et al.* [20] | 2013 |
| HPR3 | CGA/CH1271-4 | Chile | KF051917 | KF051874 | Godoy *et al.* [20] | 2013 |
| HPR3 | CGA/CH1277-17 | Chile | KF051924 | KF051880 | Godoy *et al.* [20] | 2013 |
| HPR3 | CGA/CH1277-18 | Chile | KF051923 | KF051881 | Godoy *et al.* [20] | 2013 |
| HPR3 | CGA/2978-8 | Chile | KF051925 | KF051888 | Godoy *et al.* [20] | 2013 |
| HPR3 | CGA/CH1271-5 | Chile | KF051918 | KF051875 | Godoy *et al.* [20] | 2013 |
| HPR3 | F2002 | Faroe Island | - | AF526263 | Christiansen *et al.* [16] | 2002 |
| HPR3 | NT115/05 | Norway | JN711029 | JN711068 | Plarre [25] | 2005 |
| HPR3 | ST110/05 | Norway | JN711034 | DQ108598 | Nylund *et al.* [4] | 2005 |
| HPR4 | 5T10/93 | Norway | AY853922 | - | Devold *et al.* [9] | 1993 |
| HPR4 | Gullesfjord/94 | Norway | - | AF302801 | Krossoy *et al.* [5] | 1994 |
| HPR4b | CGA/2378 | Chile | KF051925.1 | KF051882 | Godoy *et al.* [20] | 2013 |
| HPR4b | H101/04 | Norway | - | DQ108602 | Nylund *et al.* [4] | 2004 |
| HPR4c | VT11282007-35 | Chile | - | EU625666 | Kibenge *et al.* [3] | 2008 |
| HPR5 | VT11282007-38 | Chile | EU849008 | EU625667 | Kibenge *et al.* [3] | 2008 |
| HPR5 | 14/95 | Norway | AY853925 | AF364873 | Devold *et al.* [24] | 1995 |
| HPR0 | AR2/08 | Norway | FN687418 | FN687348 | Lyngstad *et al.* [17] | 2008 |
| HPR0 | AR26/08-1 | Norway | - | FN687356 | Lyngstad *et al.* [17] | 2008 |
| HPR0 | FM173/11 | Norway | JN711019 | JN711060 | Plarre [25] | 2011 |
| HPR0 | FO/01/06 | Faroe Island | - | HQ664992 | Christiansen *et al.* [16] | 2006 |

| **HPR type** | **Isolate** | | **Country** | | **Segment 5** | | **Segment 6** | | | **Reference** | **Year** | |
| --- | --- | --- | --- | --- | --- | --- | --- | --- | --- | --- | --- | --- |
| HPR6 | 25/97 | | Norway | | AY853926 | | AF364885 | | | Devold *et al.* [24] | 1997 | |
| HPR6 | 27/97 | | Norway | | AY853929 | | AF364897 | | | Devold *et al.* [24] | 1997 | |
| HPR6 | 5MR60/01 | | Norway | | AY853944 | | AY127876 | | | Devold *et al.* [24] | 2001 | |
| HPR7a | CGA/218-1 | | Chile | | - | | KJ944287 | | | This study | 2014 | |
| HPR7a | CGA/220-5 | | Chile | | KJ944289 | | KJ944288 | | | This study | 2014 | |
| HPR7a | CGA/11732 | | Chile | | KJ944293 | | KJ944292 | | | This study | 2014 | |
| HPR7a | H17/96 | | Norway | | AY853956 | | AF364891 | | | Devold *et al.* [24] | 1996 | |
| HPR7b | 26572 | | Chile | | EU449765 | | EU271682 | | | Kibenge *et al.* [3] | 2007 | |
| HPR7b | Biovac 32089P1 | | Chile | | FJ592146 | | - | | | Kibenge *et al.* [3] | 2008 | |
| HPR7b | VT11282007-37 | | Chile | | EU849006 | | - | | | Kibenge *et al.* [3] | 2007 | |
| HPR7b | Scotland 390/98 | | Scotland | | AF429988 | | AF283997 | | | Kibenge *et al.* [31] | 1998 | |
| HPR7b | 29/97 | | Norway | | - | | AF364872 | | | Devold *et al.* [24] | 1997 | |
| HPR7b | 31647-8GH | | Chile | | FJ592152 | | FJ594308.1 | | | Kibenge *et al.* [3] | 2008 | |
| HPR7b | 31648-3GH | | Chile | | FJ592168 | | FJ594334 | | | Kibenge *et al.* [3] | 2008 | |
| HPR7b | CGA/272 | | Chile | | KJ944291 | | KJ944290 | | | This study | 2014 | |
| HPR7b | CGA/302 | | Chile | | KJ944295 | | KJ944294 | | | This study | 2014 | |
| HPR7b | CGA/304 | | Chile | | KJ944297 | | KJ944296 | | | This study | 2014 | |
| HPR7b | CH01/08 | | Chile | | EU851042 | | EU851043 | | | Nylund *et al.* [4] | 2008 | |
| HPR7b | LochNevis/98 | | Scotland | | - | | AF302802.1 | | | Krossoy *et al.* [5] | 1998 | |
| HPR7b | NT81/03 | | Norway | | - | | AY973184 | | | Nylund *et al.* [4] | 2003 | |
| HPR7b | PM-4165 #8 | | Chile | | FJ592163 | | FJ594326 | | | Kibenge *et al.* [3] | 2008 | |
| HPR7f | 1508-7 | | Chile | | EU849007 | | EU849013 | | | Kibenge *et al.* [3] | 2008 | |
| HPR8 | 46/99 | | Norway | | - | | AF364896 | | | Devold *et al.* [24] | 1999 | |
| HPR8 | 48/99 | | Norway | | AY853966.1 | | AF364878 | | | Devold *et al.* [24] | 1999 | |
| HPR9 | 47/99 | | Norway | | AY853965.1 | | AF364888 | | | Devold *et al.* [24] | 1999 | |
| HPR9 | FM116/06 | | Norway | | JN711017 | | JN711058 | | | Plarre [25] | 2006 | |
| HPR9b | 13364-2006B | | Chile | | FJ592134 | | FJ594284 | | | Kibenge *et al.* [3] | 2008 | |
| HPR10 | 52/00 | | Norway | | AY853949.1 | | AF364892 | | | Devold *et al.* [24] | 2000 | |
| HPR10 | H93/04 | | Norway | | AY853948.1 | | AY973179 | | | Nylund *et al.* [4] | 2004 | |
| HPR11 | 54/00 | | Norway | | - | | AF364884 | | | Devold *et al.* [24] | 2000 | |
| HPR11 | MR71/02 | | Norway | | - | | AY127881 | | | Nylund *et al.* [31] | 2002 | |
| HPR11 | 5MR61/01 | | Norway | | AY853935 | | AY127877.1 | | | Devold *et al.* [9] | 2001 | |
| HPR12 | N5/89 | | Norway | | - | | AY127882 | | | Nylund *et al.* [32] | 2002 | |
| HPR12a | H2143/89 | | Norway | | DQ785233 | | DQ785247 | | | Markussen *et al.* [10] | 1989 | |
| HPR14 | CGA/3015-1 | | Chile | | KF051903 | | KF051855 | | | Godoy *et al.* [20] | 2013 | |
| HPR14 | CGA/3015-5 | | Chile | | KF051902 | | KF051859 | | | Godoy *et al.* [20] | 2013 | |
| HPR14 | CGA/3015-6 | | Chile | | KF051903 | | KF051860 | | | Godoy *et al.* [20] | 2013 | |
| HPR14 | FM106/05 | | Norway | | JN711014 | | DQ108599 | | | Nylund *et al.* [4] | 2005 | |
| HPR14 | Vedoy/99 | | Norway | | - | | AF302803 | | | Krossoy *et al.* [5] | 1999 | |
| HPR14 | 21/96 | | Norway | | - | | AF364886 | | | Devold *et al.* [24] | 1996 | |
| HPR15 | 5H36/98 | | Norway | | AY853958 | | - | | | Devold *et al.* [9] | 1998 | |
| HPR15 | 810/9/99 | | Norway | | EF217313 | | AF378180 | | | Kibenge *et al.* [33] | 1999 | |
| HPR15 | | Bremnes/98 | | Norway | | - | | AF302799 | Krossoy *et al.* [5] | | | 1998 |
| HPR15c | | 31682-5 | | Chile | | FJ592141 | | FJ594293 | Kibenge *et al.* [3] | | | 2008 |
| HPR15c | | 31682-10 | | Chile | | FJ592133 | | FJ594283 | Kibenge *et al.* [3] | | | 2008 |
| HPR16 | | T90/04 | | Norway | | JN711055 | | AY971666 | Nylund *et al.* [4] | | | 2004 |
| HPR17 | | MR103/05 | | Norway | | JN711025 | | DQ108606 | Nylund *et al.* [4] | | | 2005 |
| HPR19 | | 18/96 | | Norway | | - | | AF364869 | Devold *et al.* [24] | | | 1996 |
| **HPR type** | | **Isolate** | | **Country** | | **Segment 5** | | **Segment 6** | **Reference** | | | **Year** |
| HPR31 | | T121/07 | | Norway | | JN711036 | | JN711074 | Plarre [25] | | | 2007 |
| HPR31 | | T126/07 | | Norway | | JN711038 | | JN711076 | Plarre [25] | | | 2007 |
| HPR33 | | N127a/07 | | Norway | | - | | JN711066 | Plarre [25] | | | 2007 |
| HPR34 | | NT134/08 | | Norway | | JN711030 | | JN711069 | Plarre [25] | | | 2008 |
| HPR35 | | CH05/08 | | Chile | | JN711011 | | JN711093 | Plarre [25] | | | 2008 |
| HPR35 | | ST143/08 | | Norway | | JN711035 | | JN711073 | Plarre [25] | | | 2008 |
| HPR36 | | Vir22 | | Norway | | DQ785244 | | DQ785258 | Markussen *et al.* [10] | | | 2006 |

31. Kibenge FSB, Kibenge MJT, McKenna PK, Stothard P, Marshall R, Cusack RR, McGeachy S: **Antigenic variation among isolates of infectious salmon anaemia virus (ISAV) correlates with genetic variation of the viral haemagglutinin gene.** *J Gen Virol* 2001, **82:**2869–2879.

32. Nylund A, Devold M, Plarre H, Isdal E, Arseth M. **Emergence and maintenance of infectious salmon anemia virus (ISAV) in Europe: a new hypothesis.** *Dis Aquat Organ 2003*, **56**:11–24.

33. Kibenge FSB, Kibenge MJT, Wang Y, Qian B, Hariharan S, McGeachy S: **Mapping of putative virulence motifs on infectious salmon anaemia virus surface glycoprotein genes*.*** *J Gen Virol 2007*, **88**:3100–3111.
